# Welfare and Enrichment of Managed Nocturnal Species, Supported by Technology

**DOI:** 10.3390/ani14162378

**Published:** 2024-08-16

**Authors:** Fiona French, Paige Bwye, Laura Carrigan, Jon Charles Coe, Robert Kelly, Tiff Leek, Emily C. Lynch, Eric Mahan, Cathy Mingee

**Affiliations:** 1 School of Computing and Digital Media, London Metropolitan University, 166-220 Holloway Road, London N7 8DB, UK; 2 Bristol Zoological Society, Hollywood Lane, Bristol BS10 7TW, UK; pbwye@bzsociety.org.uk; 3 Zoological Society of London, London N1 4RY, UK; laura.carrigan@zsl.org; 4 Independent Researcher, Cambridge, VIC 3777, Australia; 5 Centre for Research in Animal Behaviour, University of Exeter, Rennes Drive, Exeter EX4 4RN, UK; rk528@exeter.ac.uk; 6 Faculty of Science, Technology, Engineering and Maths, The Open University, Milton Keynes MK7 6AA, UK; tiff.leek@open.ac.uk; 7 North Carolina Zoo, 4401 Zoo Parkway, Asheboro, NC 27205, USA; emily.lynch@nczoo.org (E.C.L.); eric.mahan@nczoo.org (E.M.); cathy.mingee@nczoo.org (C.M.)

**Keywords:** nocturnal, environmental enrichment, animal-computer interaction, collaborative design, potto, armadillo, aye-aye, bushbaby, coral, vampire bat

## Abstract

**Simple Summary:**

The behaviours and needs of nocturnal animals can be overlooked by humans, potentially because of our poor night vision and diurnal waking hours. Despite certain challenges in studying many nocturnal animals, appropriate provisions for their welfare should be supported in both wild and managed environments. To investigate this issue and explore ways to offer technology-enhanced welfare, husbandry practices and enrichment opportunities for nocturnal species, we conducted a multidisciplinary workshop (Moon Jam). During the event, species experts provided animal welfare briefs that related to specific challenges for nocturnal animals in different contexts. Teams of participants addressed these challenges in collaborative design sessions, producing a collection of hand-crafted models to share their ideas. An important aspect of the workshop was to be inclusive of all the stakeholders involved, including zoo management teams, animal stewards and zoo visitors, as well as the individual species. In this paper, we present our reflections on managed nocturnal animal welfare, framing these within current practices and Moon Jam workshop outputs. We contribute a set of guidelines for those involved with caring for zoo-housed nocturnal species, emphasizing the provision of technology-enhanced husbandry and enrichment opportunities.

**Abstract:**

This paper addresses the potential for technology to support husbandry and enrichment opportunities that enhance the welfare of zoo and sanctuary-housed nocturnal and crepuscular species. This topic was investigated through the medium of a multidisciplinary workshop (Moon Jam) that brought together species experts, zoo designers, Animal-Computer Interaction researchers and post-graduate students in collaborative discussions and design sessions. We explain the context through an examination of existing research and current practices, and report on specific challenges raised and addressed during the Moon Jam, highlighting and discussing key themes that emerged. Finally, we offer a set of guidelines to support the integration of technology into the design of animal husbandry and enrichment that support wellbeing, to advance the best practices in keeping and managing nocturnal and crepuscular animals.

## 1. Introduction

Human interest in other species has motivated a significant amount of research into their cognitive, behavioural and physical characteristics and wellbeing. Scientific endeavour has built a robust knowledge base describing many non-human biological traits. Some non-human animals (hereinafter ‘animals’) have been harder for scientists to investigate in their natural settings than others, because of their environment, lifestyle and associated sensory perceptions. For example, dark environments, i.e., deep sea, underground and at night [1], can be difficult to navigate and access, which may be an added challenge for researchers to obtain relevant information. People rely on human-specific sensory modalities to understand and interact with the world, which can result in information on animals that are imperceptible to human sensory capabilities potentially being overlooked without support by technology [2]. Moreover, researchers may require technological solutions to facilitate a complete conceptual picture of a dark or restricted location, whereas the inhabitants will have evolved senses that enable them to thrive in such an environment, such as adaptive echolocation in greater mouse-eared bats (*Myotis myotis*) for hunting efficiency [3]

In this paper, we focus on the lives of nocturnal species in managed environments, considering what we know of their usual behaviours in their natural habitats, and reflecting on what kinds of structural habitats and enrichment can encourage the expression of these behaviours in a zoo or sanctuary setting. 

Our method for investigating this topic involved holding a dedicated Moon Jam workshop, where participants were provided briefs from nocturnal animal welfare experts to address husbandry and enrichment challenges and were invited to respond with novel design solutions [4]. Participants included animal experts, computer science and interaction design researchers and postgraduates studying engineering and design disciplines. The Moon Jam was part of a series of multidisciplinary, collaborative design workshops (zoo jams) that provide opportunities to share skills and knowledge while discussing different themes around animal welfare in a logical, creative and open-ended way. By bringing together participants with diverse skills and shared interests in other species, the zoo jams aim to expand designers’ fields of reference and lever technology in support of welfare and enrichment goals [5]. Ultimately, the goal is to take concepts forward into action plans and evaluate them with the intended users (animal and human stakeholders).

This paper provides contextual background and offers a discussion around key topics that are perceived as being challenging for animal husbandry, with examples showing how technology can support husbandry and enrichment solutions. We share briefs from the Moon Jam to illustrate specific themes and provide topical context.

## 2. Background

Perhaps surprisingly, around 69% of described mammals are nocturnal, 20% diurnal, 2.5% crepuscular and 8.5% cathemeral [6]. Due to artificial light, humans are considered facultatively cathemeral, despite activity being concentrated during the daytime [7]. Nocturnal species evolved to rely on senses in addition to sight. For instance, some nocturnal mammals have more species-specific chemoreceptor genes and more complicated olfactory organs in comparison to diurnal mammals [8], which are responsible for smell and taste. Enhanced sensitivities may also relate to vibrations, electro-magnetic fields or air pressure. Raising awareness of nocturnal species and their specific needs is important so that humans learn how to co-exist with wildlife in their natural environments, as well as how to provide appropriate settings that support welfare in managed settings. This need is arguably increasing, where human population growth may put further pressure on resources and affect natural land use in different ways [9], for example, by reducing available habitat, introducing new dangers and causing pollution. Pollution includes not only plastic waste and chemical spills but also vibro-acoustic pollution, air pollution and light pollution. To reduce negative impacts on animals’ welfare as much as possible, we need to understand more about their lifestyles and behaviours, their modes of perception and communication and their assorted capabilities.

The difficulty of finding or watching animals in the dark can create an additional challenge in gaining knowledge on nocturnal species and their environmental and management needs. Fortunately, recent advances in remote sensing technology have provided further information from both wild populations and their natural habitats and managed animals and their artificial habitats [10]. These tools and findings can be used to optimise natural behavioural opportunities supporting improved animal welfare.

‘Enrichment’ can loosely be defined as a practice that provides additional stimuli to an animal with the aim of increasing its physical and/or mental wellbeing, and can be categorised into cognitive, food, physical, sensory and social forms [11]. The use of enrichment for animals in human care is motivated by the importance of enhancing welfare through encouraging species-specific behaviours, the need to reduce undesirable behaviours and the duty to provide good healthcare [12]. For example, a suitable intervention might offer foraging opportunities that practise skills used in the wild. This could occupy a significant portion of the animal’s time budget and mental attention, while simultaneously supporting physical objectives, i.e., body condition or weight management. Offering control and choice (some autonomy) to animals in restricted environments has been shown to reduce stress levels [13,14,15] and has other potential benefits, such as enabling the development of competence, preparing species for reintroduction to the wild and giving researchers opportunities to investigate the animals’ preferences [16]. This could lead to a better understanding of animals’ cognitive, perceptive and physical abilities, which in turn help us to consider their perspectives.

To further investigate this theme of nocturnal enrichment in managed environments with visitors, we organised a multidisciplinary workshop (Moon Jam) to explore technology-enhanced enrichment strategies for nocturnal species. We brought together a diverse group of participants, including species experts, zoo designers, Animal–Computer Interaction researchers and postgraduate students, to discuss some of the challenges faced by both animals and humans. 

## 3. Method

The workshop type used for investigating enrichment and husbandry strategies for nocturnal animals was a zoo jam, which is characterised as a multidisciplinary, collaborative design event where participants network, sharing skills and ideas. Key features of a zoo jam are (i) to have one theme with multiple associated challenges, (ii) species-specific briefs provided by animal experts, (iii) expert feedback on concepts, (iv) time-constrained activities with clear goals, (v) co-crafting and presentation of rough prototypes and (vi) open dissemination of outputs and issues raised [5].

The Moon Jam workshop [4] was held over one day in December 2023, as part of the 10th Animal–Computer Interaction Conference [17], hosted at North Carolina State University, USA. The main aims were (i) to explore husbandry and enrichment opportunities for nocturnal species through group discussions and collaborative design sessions, (ii) to address a series of animal welfare briefs provided by experts, by producing focused technology-enhanced designs for husbandry and enrichment opportunities, (iii) to incorporate animal-centred design principles, in order to extend the reach of human design and (iv) to maintain a multispecies, multi-stakeholder perspective throughout. Data collection included participant observations, feedback from species experts and analysis of the proposed designs. The methodology ensured a comprehensive evaluation of the effectiveness of these enrichment strategies in promoting natural behaviours and improving animal welfare.

The interdisciplinary nature of the Moon Jam was crucial for bringing together both expertise in animal welfare and stewardship, in particular of nocturnal species, and expertise in using technology as a means for developing complex systems to support behavioural management and to offer enhanced control of the environment, applicable to both humans and animals. Altogether, there were 18 co-located participants, comprising 6 Animal–Computer Interaction (ACI) researchers, 2 species experts, 10 postgrad students and 3 remote animal experts taking part on that day, while 12 participants (postgrad students) took part in a half-day Mini-Moon-Jam at London Metropolitan University, working with a small subset of the briefs. Some of the briefs were general, concerning challenges that face all managed nocturnal species, such as ways to provide appropriate lighting and access to fresh air. Others were specifically related to the following species: aye-ayes (*Daubentonia madagascariensis*), pottos (*Perodicticus potto*), Mohol bushbabies (*Galago moholi*), Southern three-banded armadillos (*Tolypeutes matacus*), common vampire bats (*Desmodus rotundus*) and living coral (mixed species). We explain these briefs, the associated workshop responses and the subsequent analyses in the discussion section that follows. This is presented as seven key themes: (4.1) Lighting, (4.2) Natural Experiences, (4.3) Space and Socialisation, (4.4) Foraging, (4.5) Specificity, (4.6) Multisensory Modalities and (4.7) Stakeholders.

## 4. Results and Discussion

In this section, we discuss key topics relevant to nocturnal animal husbandry in managed environments. We consider environmental conditions such as lighting and ventilation, the importance of experiencing natural events, animal behavioural needs that extend over 24 h, difficulties associated with enabling normal feeding behaviours and the potential impact of visitors in a managed environment. In addition, we consider all the stakeholders involved in a complex organisation such as a zoo, considering the educational, entertainment, research and financial sufficiency objectives that are critical aspects of a zoo’s mandate.

Each theme is introduced through one of the briefs we received for the Moon Jam, and then discussed through the lens of participants’ concepts and reflections, with expert responses.

### 4.1. Lighting 

The Moon Jam brief for mixed species of living coral, supplied by Greensboro Science Centre (GSC) in Greensboro, NC 27455 (USA), exemplifies the willingness of animal stewards to offer the best husbandry possible, as well as showing how complex it can be to provide an appropriate environment for a species that inhabits a relatively inaccessible location—in this case, the ocean floor.

#### 4.1.1. Brief: Living Coral

##### Supplied by Lindsay Zarecky and Jessica Hoffman at GSC

Most organisms utilise a variety of external cues to fulfil their biological needs. Reef organisms, like fish and especially corals, utilise both the solar and lunar cycle. Solar light is key for food production while the lunar cycle is used to determine the best time to reproduce [18].

In addition to light levels, corals are also exposed to several environmental changes including daily tidal cycles, varying levels of light (solar and lunar) intensity, seasonal temperature swings, food concentrations and periodic intense storm surges. How these environmental changes impact coral health is not well understood but could play a role in their overall health and wellbeing.

**Figure 1:**
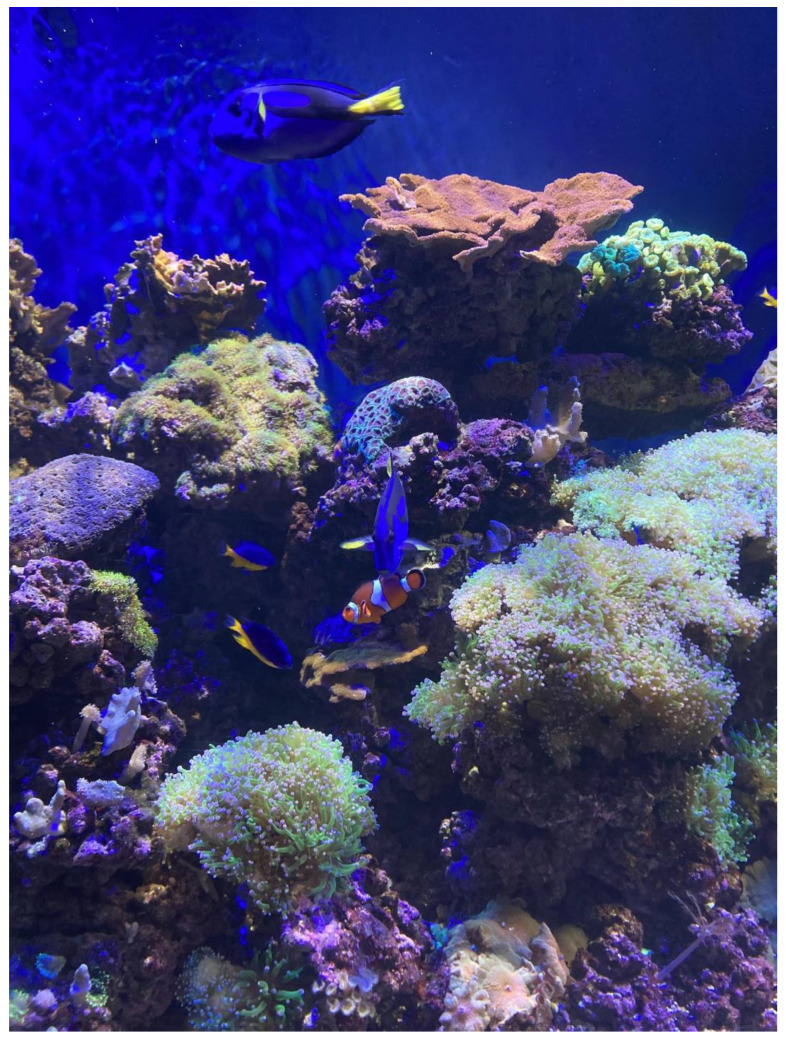
Living coral exhibit at Greensboro Science Centre. Image courtesy of GSC.

The living corals exhibit at GSC shows visitors a growing Great Barrier Reef coral reef ecosystem that includes 30+ different species of corals, coral-friendly fish and other invertebrates (Figure 1). The live coral exhibit is part of the “Communities Connected” gallery, and the tank volume is 1018 gallons (3.85 cubic metres), with a filtration system, circulation pump, heating and lighting. The substrate is crushed Aragonite gravel.

Coral health and wellbeing are important to GSC for a multitude of reasons. It is incredibly challenging to offer corals poignant enrichment. A variety of natural live and frozen foods as well as manufactured foods are offered, but it has so far not been possible to capitalise on bigger experiential enrichment that is created for other animals. In addition, replicating the corals’ natural environment to a better extent could increase the welfare and wellbeing of the corals being kept. Ultimately, the aim is to enable the corals to reproduce in captivity, thus increasing the knowledge aquarists and scientists have of a very fragile ecosystem.

Although coral care at GSC focuses a great deal on meeting their solar needs, there are not yet methods to effectively meet other environmental conditions. This brief requires teams to develop scenarios that would better mimic the environmental conditions corals experience in the wild, such as lunar light cycles and tidal shifts, without compromising husbandry needs and considering the visitor experience. Solutions should reference the environmental conditions of the Great Barrier Reef since this was the original habitat of these types of coral, but ideally, there would be some level of flexibility to adapt to Florida corals for the future.

#### 4.1.2. Responses: Living Coral

Participants found this a complex challenge, but there were several suggestions that deployed technology to address the lighting requirements of the brief. In the natural environment, the transition from day to night and vice-versa happens gradually over a prolonged period of dusk or dawn. Nocturnal animals may not necessarily live in a 24 h cycle of darkness, and creating natural sunlight patterns for them when they should be asleep will help them sense what time of year it is, as well as when it is time to be awake and asleep. In the absence of regular light exposure, such as for species living underground, the temperature can module surface emergence behaviours [19], facilitating an interrelationship with the circadian rhythm and photoperiod.

In managed environments, dimmer switches could be applied to the lighting systems of managed species, although husbandry and enrichment solutions light management should consider and value zoo staff time. A technology-mediated solution could automatically imitate natural lighting for zoo-housed species by using bulbs that can fade across different Kelvin temperatures and lux levels in order to match the qualities of the sun over the course of a day. Additionally, the photoperiod and directionality of light should also be considered to match natural conditions in the species’ home range. Feedback from zoo staff and species experts was positive on the theme of technology enabling gradual dawn–dusk lighting systems and associated changes in wavelengths. This might be a useful intervention to offer a more realistic experience to animals, which in turn from a visitor’s perspective may provide a more engaging experience allowing the observation of nocturnal animals across differing light periods.

In support of such an initiative, Moonshine is an example LED control system [19] that enables users to mimic habitat-specific conditions or certain types of light pollution. Moonshine can colour-shift LED lights to recreate natural moonlight cycles and predict illuminance in different locations during the year. Its intended use is for field ecologists and researchers investigating the effects of light pollution. However, at present, there are limitations on its wavelengths at both ends of the visible spectrum, including in the near-UV and far red, in comparison to moonlight [20], which may not be suited to nocturnal animals utilising UV vision. 

In managed environments, the effect of artificial light and what might be considered optimum requirements for nocturnal species is beginning to be investigated [21]. The colour wavelengths produced through light are important because they can influence melatonin production, a hormone concerning the circadian rhythm of species by acting as a signal to synchronise biochemical, physiological and behavioural processes, in humans [22] and non-human species [21]. Simultaneously, light pollution in wild environments creates the need to reduce disturbance to wildlife, such as through the use of amber lights, emitting no blue wavelengths, as opposed to traditional white lights which emit blue wavelengths, which may be the better choice for minimalising disruption in some nocturnal insects [23]. Since the temporal changes around light intensity, direction and wavelength are relatively predictable, many animals have evolved to use this information to regulate their behaviour. This can include seasonal adaptations, such as moulting, the timing of reproductive activities, as well as modifying food intake, activity and immune function [24].

Moreover, it is important to note that the quality of light (wavelength, intensity, duration) may all make a difference to animals in managed settings, and that appropriate light settings differ across species. More research is needed to understand and inform optimum light conditions at a species level, including their elasticity to cope outside of optimal ranges. Fortunately, through the growing application of technology in zoo research and management of animals, there are better opportunities to investigate the lighting conditions of nocturnal species in zoos. The use of artificial UV-B can be used to encourage basking behaviours of species to support vitamin D production. In fact, exposure to appropriate UV-B is now common practice for certain animals, primarily reptiles [25], and within callitrichid captive management [26]. Comparatively, less attention has been given to providing artificial UV-B to many nocturnal species that may naturally receive UV-B whilst they sleep in the wild. However, considering that the sun emits different wavelengths all working together, it is important to think about visible and infrared light along with the UV light. Moreover, the use of more natural full-spectrum light in managed environments could be utilised further to promote plant growth in nocturnal exhibits, providing more natural stimuli within indoor-only environments.

In relation to the requirement to mimic environmental conditions for mixed species of living coral, suggestions from Moon Jam participants included adjusting the water temperature dynamically using a system that tracked and automatically responded to open weather data. In parallel with this, good husbandry would try and match the current flow in the tank with live data relating to the Great Barrier Reef or Florida tides, available via the Australian Institute of Marine Science reefs portal [27] and the National Oceanic and Atmospheric Administration website [28]. The tank pump system could supply variations in nutrients with different currents, so the corals could anticipate food arrival. This would create a situation-related positive affective state in the coral, in this case, associated with having an opportunity to feed. The inclusion of such opportunities within husbandry practices is linked to the enhancement of animal welfare [29].

This introduces the second key theme that emerged, relating to natural experiences for nocturnal and crepuscular animals that are housed in indoor environments and is explored in the following section.

### 4.2. Natural Experiences 

The Fresh Air brief relates to land animals housed indoors that in the wild might receive information from moving air such as chemical signals from their conspecifics, scents relating to predators and prey, indications of food and water availability, as well as imminent changes in weather conditions.

#### 4.2.1. Brief: Air Flow 

##### Supplied by Jon Coe

Due to the typical need of day–night reversal systems to create an enclosed artificially lit environment, this might affect experiences of changing environmental conditions that are associated with outdoor air and scent sensations. Teams should think of ways to address this, considering natural experience opportunities for managed nocturnal species. 

#### 4.2.2. Responses: Air Flow 

This brief generated several possible concepts that deployed technology to enable a simulation of environmental conditions, again, mapping these to real-world conditions in the animals’ native habitats.

In general, all climate control could be managed using technology inside the enclosure, dynamically influencing factors such as humidity and temperature throughout the day and night, including providing natural gradients in microclimates. For example, artificial snow could be used to simulate winter and might be a novel and exciting substrate that encourages playful behaviour in some species. Artificial rain, mist and fog could also be provided. Conversely, basking lamps or heat sources without light such as embedded heating cables could be animal-triggered to create changing microclimates within enclosures. 

Enriching olfaction was a more challenging problem, as it required the introduction of new smells in the atmosphere—chemical signals that are often imperceptible to humans. One suggestion was to install ventilation systems that drew in naturally scented air from outside directly into the enclosure, although the use of ventilation systems is arguably more of a welfare requirement than an enriching opportunity. Another suggestion involved creating an external enclosure using breathable blackout fibres for the roof and parts of the wall, allowing air to circulate but no light to penetrate. It would also be possible, albeit expensive, to deploy photochromic ventilated glass walls, which zoo staff could control remotely, using an app. During the evening time, when the artificial light was switched on, these same features would stop light pollution from the enclosure affecting the external environment.

Since novelty is known to provoke interest in many animals, a simple low-tech idea to stimulate olfaction was the introduction of new logs or other furnishings to an enclosure. This would be likely to encourage fresh scent marking by some species. Another solution for fresh air provision that relied less on technology was to build a tunnel allowing crepuscular animals to go outside their enclosure at dawn and enter an external compound that was shared between different species at different times during the day or night. The crepuscular animals would be able to smell the scents left behind by other animals as the dew evaporated. This would also be a time before visitors arrived, so it would be quieter, and they might be less fearful. The assumption was that as the sun rose, they would naturally return to their dark setting indoors. This leads to the next important theme, around the provision of space.

### 4.3. Space and Socialisation 

This Moon Jam brief relates to the exhibiting of mixed species, providing an introduction to the advantages and challenges associated with this aspect of husbandry, and with managing space in general. Addressing the use of space within enclosures could change social behaviours within and between species, affording positive experiences.

#### 4.3.1. Brief: Pottos and Bushbabies

##### Supplied by Laura Carrigan, London Zoo

The animals in London Zoo’s Night Zone are kept on reverse lighting so their nighttime (when they are awake) is during human daytime, and then their daylight (when they go to bed) is when people have left for the day. All the animals have microchips in their shoulders.

**Figure 2:**
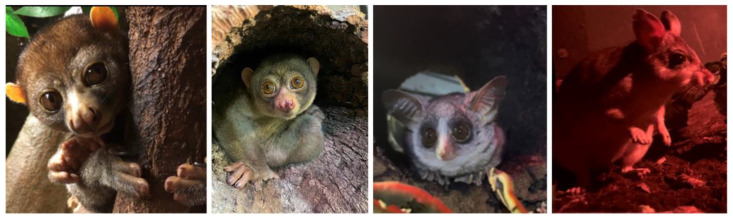
Male and female pottos, juvenile Moholi bushbaby and Malagasy giant jumping rat in London Zoo Nocturnal House. Images courtesy of Laura Carrigan, London Zoo.

Mixed species exhibits enable the maximisation of space by linking exhibits and providing natural enrichment for the animals, as well as offering a better experience for visitors as there is more activity to view. In addition to housing three Moholi bushbabies and two pottos all together, the largest exhibit in the Night Zone also includes two Malagasy giant rats (*Hypogeomys antimena*) (Figure 2).

The enclosure is approximately 3 m (H) × 11 m (L) × 5 m (W), which is about 165 cubic metres or 5827 cubic feet, with a coir/bark chip substrate and branches/liana structures throughout. Some of the structures are fixed, and others move when the animal uses them. This mixed species exhibit enables the animals to have more space compared to being housed separately, and the bushbabies have developed some form of relationship with the female potto. However, there is one issue, which is that it is difficult to control their access to food, so the older male bushbaby has gained weight. To try and counteract this, it is necessary to hand feed the pottos their favourite food (insects), because if it is scattered or placed in enrichment, the bushbabies are too quick and manage to steal everything.

Bushbabies can jump over two metres in one leap and can cover distances in seconds, which makes them much more agile and speedy than the other animals they live with. As arboreal animals, they will generally spend most of their time off the ground and traverse by jumping and running along branches. They are fed pellets, insects and gum, but they help themselves to the pottos’ vegetables as well. 

By contrast, the male and female potto move slowly across branches, although if threatened or angry, can move quickly. They cannot jump from branch to branch and will not go down to the ground for anything. The giant rat will occasionally steal live food that is scattered around the enclosure, but not enough to be an issue. Sometimes it is also necessary to hand-feed her treats like nuts and avocado since if the bushbabies like it, they will take that too.

The challenge for teams is to overcome the husbandry challenge of food provision in a mixed species exhibit by designing a feeding device that is accessible to the pottos but inaccessible to the bushbabies.

#### 4.3.2. Responses: Pottos and Bushbabies

**Figure 3:**
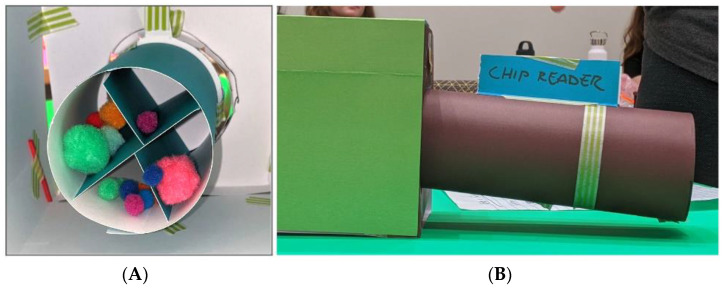
Cardboard mock-ups of tunnel system with rotating food dispenser (**A**) and higher-tech tunnel with microchip program access (**B**). Images from Moon Jam.

Two possible solutions were proposed: low tech and high tech. The low-tech version took account of the fact that pottos have longer arms than bushbabies; therefore, food could be in a space with tubular access that could only be reached by the pottos (Figure 3A). The simplicity of this design makes it very appealing. There are few parts to break, and it would be cheap and easy to place multiple versions around the enclosure.

On the other hand, since the low-tech solution was purely mechanical, it would not be able to discriminate between individuals, so could not be used for remotely monitoring access, and it would be species specific. Also, it would not dispense food over time, meaning that care staff would always need to be involved in provisioning. The higher tech solution required power and electronics but had more functionality and would be flexible for different mixed species enclosures. It could be set with a timer to dispense different food items and could discriminate between micro-chipped individuals (Figure 3B), enabling a clear record of which animal accessed which kind of food at what time. This solution also used the long tunnel concept to restrict bushbaby access. 

Alternatively, bushbabies could be lured to their feeding portal and held inside while pottos had time to forage. However, this concept would be more complex to build and potentially less practical for other exhibits. Other proposals included setting aside separate smart-gated spaces in the enclosure for food delivery, with the gate operated by reading animals’ microchips [30,31]. A problem with this idea was that the bushbabies could jump nimbly through the gate when opened by a potto. Nonetheless, in general, the concept of animal-controlled ‘smart gates’ that facilitated the animals’ ability to freely access areas within an enclosure, using a microchip-controlled system, was regarded positively by zoo designers and staff.

Enclosure area standards are an important benchmark, and it was noted that existing guidelines represent minimum standards. The clear trend is to enlarge these areas in zoos and related facilities, especially as animals can become more physically fit and intellectually motivated. Broad advice from experts is to aim to exceed today’s standards and best practices. At the Moon Jam, the recommendation was to use space creatively and with regard to natural behavioural patterns, aiming to maximise and optimise what was available to use by the animal. One example was to have maximum rather than minimum branching cover for climbing/arboreal species, thus providing a wider surface area for spreading out food or food devices to promote exploration and foraging. In general, the more complex the environment, the more opportunity there is to include branching routes and structures that support species-specific mating, scent marking and social and toilet-related behaviours. 

While it is possible to develop enriching features within existing enclosures, there are many opportunities that can be offered in the design of new enclosures as well. A single space or area might not represent the best design, although combining exhibits can offer new challenges, as demonstrated by the mixed species enclosure housing pottos and bushbabies. Having the choice, control and ability to move from place to place is enriching, especially if each place offers complimentary resources. Long connections between different accessible enclosures (trailways) can also provide interest and stimulate exploration. The Center for Great Apes in Florida (USA) constructed one of the first of these for their primates, and the concept has been taken up by other zoos. An ambitious example is Philadelphia Zoo’s Big Cat Falls Trailway which allows the felids to walk over visitors’ heads as they travel to different but connected spaces [32]. It gives the animals a rich perspective on the environment around them while protecting other species from predation. Such features can also be popular with visitors. While these examples were developed for diurnal species, enclosed indoor trails or flyways (either for single or mixed species) could be developed for animals housed in large nocturnal houses.

A potential complication associated with rotating or alternating species through enclosures is the risk of bacterial or viral transmission from one to another. Albeit, these risks could be reduced through regular animal health faecal screening methods. In addition, staff should wear sterile boots and gloves to clear enclosures and handle animals. In cases where infections are confirmed, zoos may increase their biosecurity practices, which extends to limiting the use of mixed species exhibits. This issue potentially exists within any open enclosure, because if the carrier was a wild mouse or bird, droppings could be present in any accessible browse. 

Despite some challenges, particularly in enabling allocated food provision for species, carefully monitored mixed species exhibits can provide food and sensory-based enrichment without the occurrence of intra or interspecies conflict [33]. The following briefs also incorporate food provision, with a focus on natural foraging behaviours. 

### 4.4. Foraging 

Nearly all the Moon Jam briefs are related in some part to feeding—access to food, behavioural repertoires associated with foraging and hunting and managing change, such as age-related conditions. 

Food-based enrichment can support natural feeding opportunities and increase species-specific behaviours in acquiring food resources. For instance, burying food or placing food in Kong pet store toys could generally increase the frequency of digging and rooting behaviours. Specific examples in nocturnal species include the use of artificial termite mounds increasing sit-and-wait predation behaviours in bushbabies [34]. Promoting foraging behaviour is arguably important for species to take control over their feeding experiences, which is likely to be intrinsically connected to their wellbeing and survival. Certainly, in the wild, predator species can incur a risk of mortality by starvation [35,36], which may be increased without the skills to acquire food or an opportunity to express them. If species in managed environments are to be considered for wild release programmes, which may be a growing priority for zoo organisations and rehabilitation centres in the future, behaviours directly associated with a species’ ability to secure food in the wild should be strongly encouraged in managed care. As a case in point, abnormal dentition, gouging behaviour and frequency were reported in a translocated Javan slow loris that was later found deceased with a deformed jaw [37], indicative that these deviations in feeding behaviours may have contributed to its mortality.

The following brief from North Carolina Zoo illustrates some of the challenges associated with enabling natural feeding behaviours for nocturnal species kept in managed environments.

#### 4.4.1. Brief: Common Vampire Bats

##### Supplied by North Carolina Zoo 

North Carolina Zoo is home to a colony of 66 common vampire bats, which live in a nocturnal cave area. The habitat is on a reverse light cycle, creating nighttime lighting conditions for the bats during daytime hours, which encourages wakeful periods for the bats during operating hours. 

Common vampire bats are considered sanguinivores, or animals that consume only blood. They are the only species of bat that only consumes mammalian blood, and in a natural habitat, feed from a wide range of sleeping mammals. Using echolocation, along with specialised scent detection, vampire bats find the warmest spots on the mammals, where blood runs closest to the skin. The bats typically land near their prey and then walk and climb to the best feeding location. A small hole (approximately 0.5 mm) is punctured with their sharp teeth and the anticoagulant contained in their saliva ensures blood flow. This process can take around 20 min but rarely wakes the prey animal. Vampire bats can consume about 2 tablespoons (35.5 mL) of blood at a time and must eat at least every other day. 

**Figure 4:**
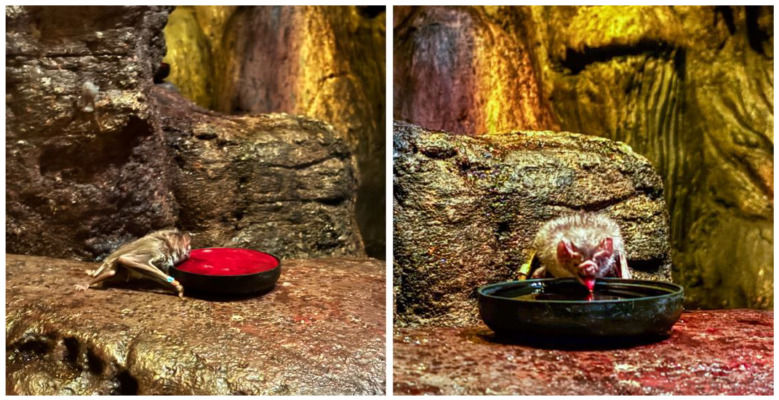
Vampire bat feeding on cow’s blood. Image courtesy of the North Carolina Zoo.

At the North Carolina Zoo, vampire bats are fed harvested cow blood (Figure 4). The blood is treated to reduce coagulation and stored in the fridge to keep it fresh. Although this is a common management practice, it restricts how blood can be presented to bats. First, the blood cannot be warmed before presentation, as this would reduce its quality and duration of freshness. Second, the blood is usually placed in open dishes. These factors both reduce the natural hunting and feeding behaviours of vampire bats.

The challenge for teams was to design a system for feeding that supports the expression of the bats’ natural foraging behaviours.

#### 4.4.2. Responses: Common Vampire Bats

**Figure 5:**
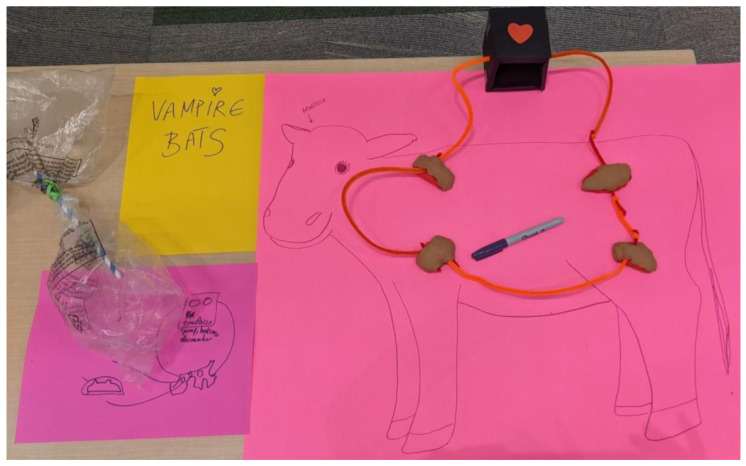
Physical mock-up of compression system comprising blood bags holding cow’s blood. Image from Moon Jam.

The main concept from participants involved creating an artificial system for presenting fresh, warm blood that circulated between a set of blood bags (Figure 5). The bags would be made from a biocompatible polymer so the bats could pierce them safely—similar to the natural casing used on sausages. More research would be required to test whether the bats could smell the blood through this artificial skin and to find an appropriate method for maintaining suitable pressure within the system. 

Animal care staff found this concept to be very creative and reflective of vampire bat behaviour in the wild. 

Food-based enrichment can form part of the overall healthcare management of animals such as by increasing activity budgets, though these effects can differ between species [38]. For nocturnal reptiles, examples of enrichment include stimulating the olfactory senses and associated behaviours of tongue touching, lip-licking and sniffing in leopard geckos [39]. Mimicking wild food presentation may also be important to improve food consumption, such as manual shaking of mice to encourage a strike response in snakes. Hanging up meat could also encourage more active and extended feeding behaviour durations, which may be important in species prone to obesity and muscle wastage, or for species that otherwise spend a large amount of their time in resting behaviours [40]. 

Visual, olfactory and auditory senses may also be stimulated through the enrichment design (materials, size, shape, complexity). The enrichment could simply be a novel food source and/or changes in its presentation, such as the use of live fish, which has been demonstrated to reduce pacing in tigers [41]. Moreover, by increasing the diversity of invertebrate food sources, different feeding behaviours can be promoted, such as digging or object manipulation to obtain burrowing mealworms, compared with the need for running, climbing and catching to capture locusts or crickets. 

Another aim for food-based enrichment may be to help reduce undesirable or stereotypy behaviours, such as excessive pacing in felid species [42] or stereotypy swimming patterns in Vietnamese pond turtles [43]. More research is needed to investigate any physiological responses to enrichment, including food-based enrichment. Nonetheless, preliminary evidence has identified reduced faecal corticosterone following combined enrichment use (manipulable, sensory, and feed) in Asiatic lions [44], suggesting that in this case, the enrichment had lowered stress hormones within a captive environment.

Notably, enrichment can be manipulated to increase the level of difficulty, depending on whether the desired goal is to extend the duration of activity, to suit individual considerations (age and health status) or to address individual response variability to enrichment [45]. This highlights another important theme, which is the provision of specificity—solutions for individual members of a species. As with humans, there is rarely a ‘one-size-fits-all’ answer that works for everyone.

### 4.5. Specificity 

The next Moon Jam brief featuring food provision illustrates the potential need to tailor husbandry practices and enrichment for a specific individual depending on their circumstances. The challenge of attending to the welfare of a particular animal involves learning about individual as well as species characteristics, to provide a personalised experience for that animal. Designing enrichment for one individual can give rise to insights pertaining to all members of that species, as well as to other animals experiencing similar life conditions.

#### 4.5.1. Brief: Aye-Aye

##### Supplied by Paige Bwye

**Figure 6:**
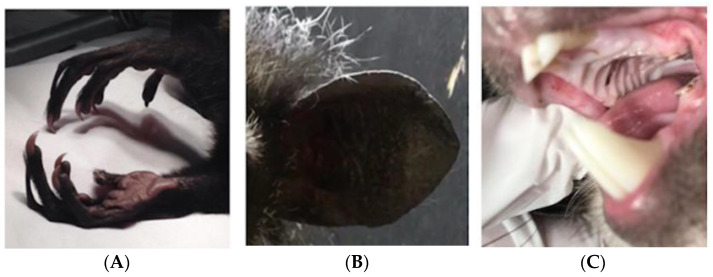
Aye-aye features—hands (**A**), ears (**B**) and teeth (**C**). Image courtesy of Paige Bwye.

Aye-ayes are a nocturnal lemur from Madagascar possessing unique adaptations including their large ears, their thinner and elongated middle digits on each hand, and their overbuilt masticatory apparatus [46], to assist specialised tree-gouging behaviour. As percussive foragers, aye-ayes use their specialised middle digit (Figure 6A) to tap on bark while thought to be listening to vibrations within their auditory range (Figure 6B), indicating invertebrates inside. Once identified, they gnaw into the bark to access their prey with their continuously growing incisors (Figure 6C). Using their middle digits, they then excavate the prey deep within the hollow crevices. When they are not eating insects, they also forage on the ground for seeds and fallen fruits.

For the purpose of the brief, the aye-aye enclosure dimensions to consider are 6 × 4 × 6 m (approx. 150 cubic metres or 1766 cubic feet), featuring horizontal and vertical branching, elevated nest boxes and rotten logs at ground level. Aye-ayes are generally destructive in captive environments because they can chew through strong surfaces, rendering many enrichment materials unsuitable. Moreover, they are a socially dispersed species and typically housed solitary in zoos.

**Figure 7:**
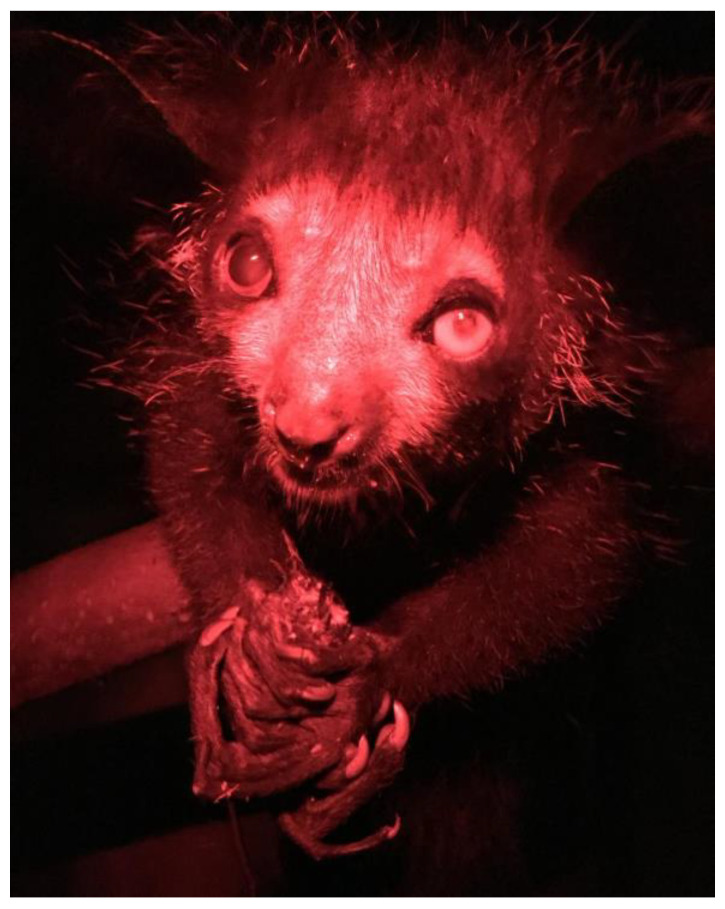
Aye-aye with cataracts. Image courtesy of Paige Bwye.

The aye-aye used for this brief is beginning to show age-related changes, including cataracts in both eyes (Figure 7). Although his mobility is good, he cannot see individual insects in his environment. For this reason, it would benefit him to make his food provision easier to access to ensure consumption, but overtime this could lead to his teeth overgrowing from reduced gnawing opportunities. Teams were asked to apply what they had learnt about aye-ayes and this particular individual’s background information into consideration to produce a technology-based solution that promotes species-specific foraging and incorporates dental health (serving as enrichment, but also as a required husbandry intervention).

#### 4.5.2. Responses: Aye-Aye

The simplest, non-technical concepts produced by teams were chew toys made from hard hollowed wood, such as bamboo, filled with termites and sawdust, then sealed at the ends, so the aye-aye would need to use his teeth to access the food. The lengths of wood would be secured in a log holder. An alternative, more proactive suggestion was to use a scented coconut shell to hold food, since the shell would be abrasive and wear down his teeth.

**Figure 8:**
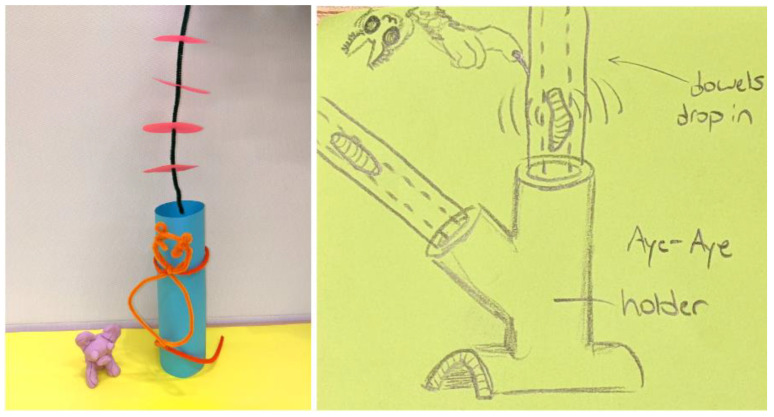
Two versions of a food holder—removable plate system and hollow dowel. Images from Moon Jam.

Teams spent a significant amount of time designing a reusable feeder that could track bite activity. This concept used flat plates of hardwood inside a tough edible tunnel that the aye-aye had to bite, so the tunnel was disposable, but the centre part could be refilled (Figure 8). Insects were delivered on the plates and a sensor placed in the central section could measure bite activity. In a situation where the aye-aye could not hear the insects, a vibromotor would be used to simulate the sound of moving food; alternatively, it would be possible to amplify the sounds of insects, so they were easier to find. Expert responses identified that the use of any sensor materials would have to be inaccessible to the aye-aye directly to prevent the consumption of non-edible material.

For species that require regular mastication good husbandry practices should seek to provide food-based enrichment which enables gnawing, which may contribute to preventative and reactive dental care. For instance, the provision of hardwood sticks resolved malocclusions by improving molar occlusal wear in pine voles [47]. While the teams’ enrichment concepts worked towards meeting the dental health goal that was set in the brief, the motivation to forage and feed might not have been fully stimulated if the visually impaired aye-aye had been presented with a concealed pipe containing insects. However, as his hearing is unimpaired, acoustic enrichment could complement food-based enrichment in this scenario using technological ‘lures’ in the form of insect sounds in different locations around his enclosure to encourage a foraging response.

This leads us to the next theme, which relates further to foraging solutions taking into consideration the sensory modalities used by another nocturnal species.

### 4.6. Multisensory Modalities 

As mentioned in the Background section, nocturnal species do not typically rely on vision, but also use acoustic, olfactory and tactile signals, and may have the ability to perceive their world using other sensory modalities such as sensitivity to electromagnetic fields, humidity and air or water pressure changes. The Moon Jam brief that introduces this topic focuses on the foraging behaviour of an armadillo. 

#### 4.6.1. Brief: Southern Three-Banded Armadillo 

##### Supplied by Robert Kelly

**Figure 9:**
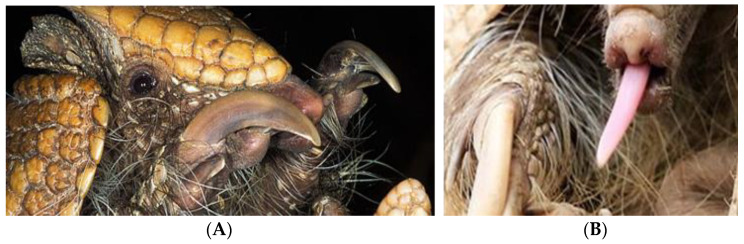
Armadillo claws (**A**) and tongue (**B**). Image courtesy of Robert Kelly.

The southern three-banded armadillo is a nocturnal species native to Central South America, inhabiting savanna and dry forest. They can use their distinctively long and powerful claws (Figure 9A) to dig through tree bark and termite mounds to forage for insects. Like their anteater relatives, these armadillos possess long and sticky tongues (Figure 9B), and shovel-like snouts to extract termites from small crevices and to root around in the forest floor for other insect prey. Due to their nocturnal traits, this species compensates for poor eyesight by being equipped with well-developed auditory and olfactory senses. 

Despite being a commonly housed zoo animal, armadillos are comparatively understudied. They are primarily solitary but may often be housed in breeding pairs in the zoo. Heated nest-boxes are normally provided as sleeping quarters and refuges. Armadillos are typically housed in reversed day–night systems to promote activity levels, although differences amongst species exist. Variation in ambient temperature influences levels of activity in armadillos. For example, lower air temperature in the southern three-banded armadillo is associated with decreased activity, whereas the inverse is observed in the six-banded armadillo (*Euphractus sexcinctus*), which becomes more active with decreasing temperatures [48].

As a terrestrial species, substrate provision is an important consideration. Typically bark chips are used which can promote natural digging and rooting behaviours. However, this means that smaller enrichment devices can sometimes be displaced or accidentally dug beneath the substrate. Armadillo claws are very powerful, and they can be destructive animals—they have the potential to dig through small, discrete gaps and are strong enough to excavate concrete, so teams should bear this in mind when considering construction materials and device design. Being accommodated in darkened environments, and due to their poor eyesight, armadillos may struggle to locate enrichment without some form of olfactory cue. 

**Figure 10:**
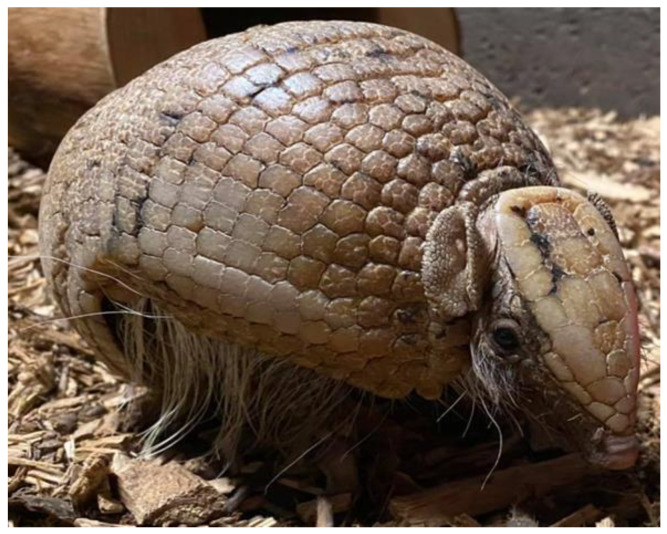
Geriatric southern three-banded armadillo. Photo courtesy of Robert Kelly.

An adult male southern three-banded armadillo is currently housed solitary at Amazon World Zoo Park on the Isle of Wight (Figure 10). He occupies an enclosure of 34 cubic m (1200 cubic feet) with a coarse bark chip substrate. Logs are spaced randomly around the enclosure with a heated nest box at either end. Due to his old age, his eyesight is very poor, and he is particularly sensitive to loud noises and vibrations. Can teams devise an idea that promotes foraging activity, taking his age and requirements into account?

#### 4.6.2. Responses: Southern Three-Banded Armadillo

**Figure 11:**
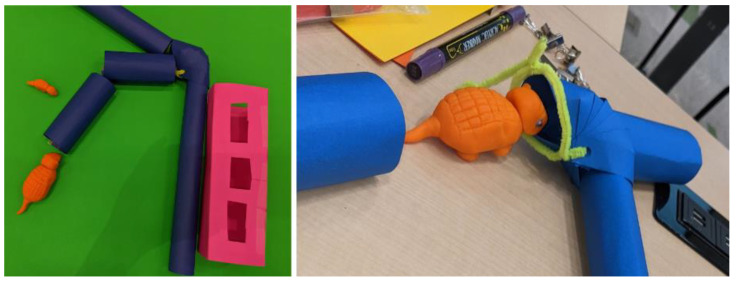
Model of the armadillo investigating a tunnel system with an adjacent play tunnel. Images from Moon Jam.

Since armadillos spend the majority of their time underground, this concept involved a modular raised tunnel system (Figure 11). The sections were joined with junctions, so staff could alter the routes periodically to make it more dynamic. There would be puzzle food boxes located at junctions, easy to fill from above. In theory, the armadillo would be able to smell which ones had treats, using his tongue to reach the food inside the containers. Sensors could monitor his behaviour and light up LEDs on the top of the tunnel as he passed so that visitors knew where he was at any given moment. Another possibility would be to build the tunnel from clear acrylic so people could watch in low light. This assumes that if the individual cannot see very well, the tactile and physical character of the tunnel would reassure him. 

A solution to lengthening claws due to inactivity could also be to coat the architectural flooring surface with an epoxy-bonded abrasive grit such as ‘stonhard’ flooring beneath the bark mulch. To promote activity levels in zoo-housed armadillos and encourage animals to engage in species-specific behaviours, husbandry and enrichment programmes have been devised, but not consistently put into practice for a number of reasons, including complex organisational policies, excessive caution and difficulties judging effectiveness [49]. A comparison of the nine-banded armadillo (*Dasypus novemcinctus*), the Llanos long-nosed armadillo (*D. sabanicola*) and the southern naked-tailed armadillo (*Cabassous unicinctus*) suggests that food-based enrichment may be ineffective at changing armadillo activity periods, but is able to reduce abnormal behaviour and increase foraging behaviour [50], although further research using larger sample sizes is required to confirm this. Similarly, food-based enrichment was considered ineffective at promoting activity in the six-banded armadillo (*E.sexcinctus*), the large hairy armadillo (*Chaetophractus villosus*), and the southern three-banded armadillo (*T. matacus*) [51]. Armadillos compensate for poor eyesight with keen olfactory senses and can respond and discriminate between different scent-based cues [52]. Thus, scent-based elements could be considered within the enrichment design.

The Moon Jam brief for the armadillo generated a lot of ideas around potential solutions that could simultaneously engage zoo visitors. One idea involved a perforated tunnel wall that ran adjacent to a section of his tunnel—aimed at encouraging children to enter the darkened armadillo world, where they could both smell each other, for mutual olfactory stimulation. However, criticism of this was that ‘parallel play’ often results in loud, disruptive behaviour by participating children, who become lost in their own interactions, and, therefore, miss the educational value of the experience.

Other physical games included keeping completely still in a specific location for a short duration, to trigger a display of live infrared camera footage and various acoustic games that involved intense listening. There were also ideas for potential mobile or touchscreen games, such as armadillo maze puzzles and ‘Spot the Armadillo’, which involved trying to guess his location correctly as he moved along the tunnel system (when the LEDs dimmed). The computer games were all apps that could be developed without introducing anything novel to the armadillo enclosure, as a way of maintaining visitor engagement. 

The hearing capability of armadillos is thought to be sensitive, and, currently, we have a limited understanding of their responses to auditory stimuli in captive environments. Therefore, acoustic enrichment would be a useful area of research to develop. Human sound pollution presents a different challenge within managed environments. Special design and construction materials are required to lessen external auditory disruption that might be caused by loud zoo visitors, ventilation and pumping equipment, after-hours concerts or occasional nearby construction projects [53,54,55]. Zoo-housed armadillos can be susceptible to stressors including increased handling for education purposes [56] and visitor presence.

We argue that more attention should turn to understudied nocturnal species, such as the armadillo, to ensure that husbandry and management practices to promote welfare can be established. This involves the cooperation and collaboration of zoo staff, managers, stewards, designers and researchers, and ideally would raise awareness of these animals to the wider public through visitor engagement. The following theme speaks to the importance of taking all stakeholders into account (humans and animals) when undertaking design projects.

### 4.7. Stakeholders

Developing artificial habitat design solutions requires an in-depth knowledge of the context, which in the case of zoos involves understanding the requirements and perspectives of staff (managers and stewards), visitors, designers, researchers and non-human animals. Moon Jam participants primarily focused on the animal briefs they were given, acknowledging after the workshop that they had considered nocturnal husbandry and enrichment goals and more-than-human aesthetics, notably sensory modalities, cognitive and physical characteristics, and social and environmental preferences of the species in question. In addition, they were tasked with producing a focused, feasible design that considered context (physical, cultural, geographical, social environment, time constraints and ease of use for human carers), logistics (skills and resources required, financial and time implications), zoo mission statements (education, conservation and entertainment objectives for visitors) and research potential (including design evaluation, iteration and testing with users, and publication). 

The authors concluded that the length of the Moon Jam event precluded such a detailed analysis of the context for each brief, but that these important considerations would be part of future plans when moving forward from conceptual design to prototyping.

In relation to zoo management, the viability of enrichment should be assessed in terms of inputs (e.g., cost to build and maintain, including staff time) and outputs (e.g., the measurable effect on the species or individual, subsequent visitor engagement, opportunities for publicity around welfare, research dissemination and contribution to wildlife initiatives). The challenges, opportunities and needs of researchers can be supported through access to technology such as infrared video, tracking and recording devices [10], as well as developing their ideas for experimental design. Animal stewards are crucial to the success of any project since they are the fundamental link between humans and other species; they implement initiatives, collect data, interpret affective states and offer feedback on systems, as well as undertaking their usual caring responsibilities. They should therefore be included as contributors, along with animal carers, designers and researchers to any enrichment design discussion. 

As mentioned earlier, visitor engagement is critical for a number of reasons: (i) it facilitates access to animals so that people better understand other species that share our world; (ii) it increases the footfall required to maintain the zoo as a viable financially independent organisation; (iii) it supports the funding of research and conservation projects and (iv) it can indirectly contribute to advertising since people will share their experiences on social media.

The Moon Jam brief Stop the Flashes aims to identify alternative schemes for supporting the visibility of nocturnal species in day–night reversal to visitors, which excludes the use of visitors’ personal flashlights. 

#### 4.7.1. Brief: Stop the Flashes 

Balancing the lighting needs of visitors and nocturnal animals under day–night reversal can create a challenge in managed environments which could amplify the use of visitors using phone flashes/flashlights. In some incidents, visitors may turn on their own lights to be able to move around more easily in dim light areas and we must acknowledge that not all visitors’ eyesight will be the same as one another. In other cases, visitors could be tempted to use their phone lights to improve the visibility of species and proceed to take photos with flash, failing to understand the negative implications of such bright lights. It is important to balance the lighting needs of the visitors for safety and experience satisfaction whilst maintaining species’ requirements for appropriate lighting and light cycles, which are critical for their biological rhythms, contributing to health and wellbeing.

#### 4.7.2. Responses: Stop the Flashes

Participants drew on a combination of technological intervention and gamification techniques to dissuade visitors from using their phones. Two main concepts were suggested, the first of which involved installing special photochromic glass viewing windows with controllable properties. A bright light would trigger the window to go dark, simultaneously stopping everyone from taking photos. The use of peer pressure to stop people from being antisocial was deemed to be more effective than notices, so this concept worked as social engineering. This is an example of gamification techniques being deployed to manipulate human behaviour and was supported by zoo design colleagues. 

The second idea was to develop an app that visitors could install on their phones. It would have access to the phone settings (such as location-based tracking) so it automatically restricted flash usage when they were inside the nocturnal house, but it had exciting benefits too. For example, visitors could access remote low-light cameras to observe animal activity from inside the enclosure on their phones or pads. 

Visitor experience could also be enhanced if humans were able to transition more gradually from daylight to the interior of a nocturnal exhibit, by passing through a darkening corridor that enabled their eyes to adjust normally. One possible solution to extending the visitor eye adjustment time would be to organize the nocturnal species encountered, such that the route began with the most light-tolerant species and progressed to the species needing the darkest environments. Another way to accommodate human eye adjustment to darkened nocturnal areas in new constructions is to locate nocturnal exhibits connected to indoor mid-light level facilities such as reptile houses or museum areas. Visitors gradually adjust to mid-light levels while viewing reptiles, then adapt to lower light levels viewing nocturnal species. Returning via the mid-light reptile displays allows visitors to readjust their vision before exiting to outdoor sunlight levels. Alternatively, staff, interactive activities, or videos could be strategically placed at the beginning of exhibits to increase visitor time in adjustment zones. Ultimately, when nocturnal exhibits are designed to incorporate animal welfare, theming and visitor experience, attraction ratings are likely to reflect this. An example is the 85.2% ‘very good’ visitor rating of Singapore’s night safari, an attraction dedicated to nocturnal species, exceeding ratings over ten other nature-based attractions in Singapore [57].

It is also important to consider how to handle visitor expectations, so staff need to find ways to be clever in how to offer engagement with nocturnal species whether that is through allowing visitors to view species across artificial sunrise and sunset times when these animals are more visible as previously discussed, by providing animal talks on these species from a trained member of staff who is able to locate the animal, or using video screens to show the behaviours of these animals that may or may not be live footage (such as cameras placed in view of enrichment).

## 5. Guidelines for Nocturnal Enrichment

This section offers a brief overview of the technology-enabled ideas we have explored that can support the wellbeing of nocturnal species, and some guidelines for developers.

### 5.1. Overview 

**Figure 12:**
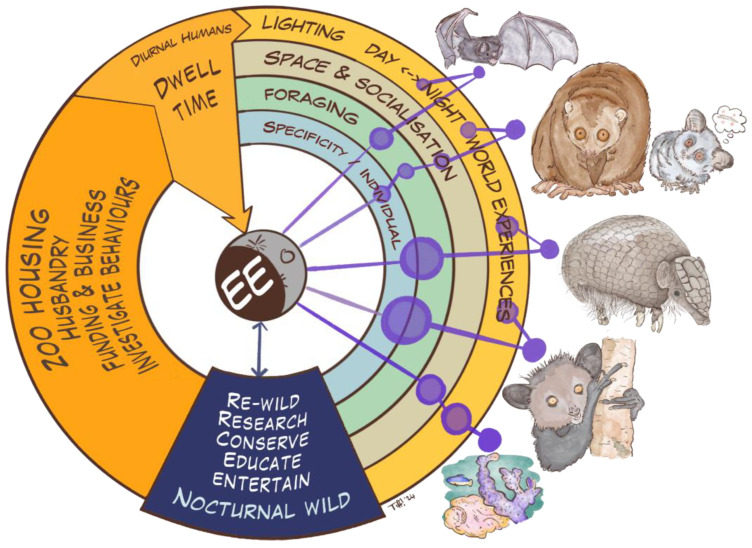
Husbandry and enrichment design framework for nocturnal species. The purple circular points correspond to the focal points of each species’ brief (the larger the size, the more targeted the brief was to the consideration). Image designed by Tiff Leek.

The examples in the Moon Jam briefs (Figure 12) and associated responses in the Discussion section have illustrated how nocturnal species rely extensively on tactile, olfactory and auditory senses, are highly sensitive to variations in wavelength, intensity and direction of light, and can perceive fluctuations in heat, humidity, pressure and direction within the medium they inhabit (air or water) and also through their substrate and other environmental features. There are also other senses available to non-humans but apparently undetectable by humans, such as perception of electromagnetic fields, and senses not previously mentioned, such as taste and proprioception. 

In relation to lighting, technology can enable auto-transitions across day and night, simulating real-world conditions. Sunlight and moonlight can be emulated, with wavelength, angle and duration corresponding to species-specific global locations. Photochromic glass can be controlled to achieve different effects, from masking light to masking the presence of visitors on the other side of a barrier. In these scenarios, the technology supports both stewards and animals, by automating procedures to save human time and by using sophisticated techniques to recreate natural experiences. Other environmental changes, such as ocean tides and weather variability, can also be recreated using technology, to support realistic behavioural responses from the animals involved. 

Tech can also be used to sense and track animals’ use of space, through the use of radio-frequency identification (RFID) with tagged individuals, while gated systems can limit or permit access to certain locations. Sensors could, for example, reveal the whereabouts of animals underground to visitors, using lights above a tunnel, or through streaming infrared camera footage. Many species have round-the-clock behavioural needs, so cameras could be used to monitor activity budgets over a 24 h period.

In relation to foraging, devices can release food randomly, provide specific nutrition for particular individuals or be programmed to be triggered by both animals and humans. Tech would also enable the development of a system that mimicked live animals, to feed warm blood to bats, and the generation of artificial acoustic signals to lure animals to different resources and encourage activity. Many animals have individual needs, and technology can also be used to monitor their health, such as capturing data about bite strength or body temperature. The Moon Jam briefs did not include nocturnal avian and reptilian species, however, the ways in which technology can support the welfare of nocturnal species can apply to these animal groups.

### 5.2. Multispecies Interaction Design 

It is important to note that animals co-evolved with environmental conditions directly affecting their welfare, by influencing their evolutionary strategies for survival and reproduction, notably feeding, social interactions and exploratory behaviour. Moreover, all living organisms experience and make sense of their world through their sensory perceptions, and many have sufficient autonomy to enact choices based on what they perceive. The opportunity to make meaningful choices within complex and manipulable artificial habitats offers managed animals control over their life experiences, which is central to animal welfare [58] and is, therefore, an important aspect of an enrichment plan. While some reactions may be ‘hard-wired’ (driven by evolved instincts), others may be based on individual preferences (e.g., favourite treats) or neurological reinforcement that has been established through previous experience (e.g., positive or negative reactions from conspecifics). In the wild, animals have the ability to choose between a wide range of different resources and experiences including microclimates, light levels and many more. They should have access to similar choices while in managed care environments.

The ability to perceive phenomena, discriminate and choose between different sensorial experiences and make these decisions based on previous and immediate personal experience is indicative of aesthetic sensibility [59,60]. This suggests that environments, resources and interactions with others can be more or less pleasurable for animals, depending on their perceptions and preferences. We therefore argue that designers should carefully consider species-specific characteristics in relation to the aesthetics of system design when developing new features or experiences for enclosures. This includes features such as the smell, taste, texture, malleability, colour, shape, sound, position and interaction associated with any device.

Games, toys and control systems with species-specific interfaces offer opportunities for cognitive and sensory stimulation to animals within managed environments, as well as autonomy and the chance to gain competence [61]. This applies to both humans and non-humans, providing zoo staff with interactive features they can control, visitors with engaging apps that educate in entertaining ways, researchers with ways to investigate animal preferences and capabilities and the animals with different means to learn new skills, make relevant decisions and work for rewards (contra-freeloading) within their enclosures.

Aside from feasibility and cost, key considerations to be made around the use of technology are related to (1) Ethics, (2) Messaging, (3) Usability, (4) Teamwork, (5) Futureproofing, (6) Biocentric design, (7) Evaluation and (8) Contextual relevance and broader applications.

1. Ethical issues are complex and a discussion of the many perspectives on animal welfare and management is beyond the scope of this paper. We point to the field of Animal–Computer Interaction, where there are many examples of literature that defines, and projects that exemplify animal-centred design principles. Examples include descriptions of design methodologies and frameworks that enable animals to be involved in the design process as contributors [62] and discussions around values beyond welfare, ergonomics or usability, such as privacy and consent [63]. Ultimately, it is the design team’s responsibility to find ways to communicate the team’s intentions with client animals and to interpret the animal’s resulting responses.
2. Messaging relates to direct visitor–animal experiences as well as to signage and apps for zoo visitors, which can give a powerful signal about the attitudes and priorities of the establishment. For example, do the display techniques demonstrate human dominance over animals and the environment, or represent humans and animals as equally entitled residents of Earth? Could animals, apps and games be mistaken for human children’s entertainment? Does the signage empower or trivialise human endeavours to support animals’ lives or desires?
3. Usable systems are a fundamental requirement, whether being used by humans or animals. For humans, technology needs to be easy to learn and use. For non-humans, it is crucial for designers to gain a deep understanding of the species’ natural history and an individual animal’s personal history. With such knowledge it should be possible to lever the animal’s usual behaviour—the affordance of the system should include mechanisms, presentation, and aesthetic qualities. Being able to work with prototypes is essential, so as to iteratively test designs and modify them based on the animal’s actions and reactions.
4. Simplicity in design is desirable but can be hard to achieve, and it may seem easier to rely on known technological solutions. Often, however, collaboratively working on challenges within a multidisciplinary team can generate simple, non-technical solutions that are cheap and easy to implement, such as the ‘potto sleeves’ that restrict bushbaby access to treats. Teamwork offers participants ownership of the design since everyone participates in its creation; this, in turn, is motivating for stakeholders and facilitates future deployment.
5. Futureproofing involves ensuring that technological solutions do not quickly become obsolete. It is appropriate to design flexible and adaptable systems that can easily be maintained or adjusted, provide support for human users and be willing to make changes as new knowledge becomes available or enclosures are updated. This concept also applies to advocacy around welfare and enrichment, meaning that today’s ‘best practice’ may be considered a very low threshold in the future.
6. It is important for humans to accept that we do not know everything about other species. The state of knowledge we have today will inevitably be superseded in a few years, and corresponding welfare standards and practices for managing animals will also change. As it stands, biocentric design is the optimum approach for creating zoo enclosures—in other words, trying to recreate the environment in which the animal evolved, with as many of the relevant experiential features as possible. This involves the human design team trying to ‘see the world with new eyes’, which may involve the use of technology to expand our limited perceptions. Immersion in the umwelt of another species is an exciting prospect that can give researchers and designers insights that transcend their original context and facilitate the development of new knowledge and opportunities to better understand our ecology.
7. Feedback from Moon Jam participants suggested that there is a lack of available information on suitable methods for evaluating enrichment designs. Planning a research study requires viable and specific research questions. For example, we cannot ask: ‘Is it successful?’ about a new enrichment device without first defining our measure of ‘success’. Traditional scientific papers are heavily biased towards collecting and interpreting quantitative data, but there is a growing appreciation of qualitative research, particularly in the early stages of a project, for identifying and refining problems, and later on, to collect stakeholders’ perspectives, for example. Data does not need to represent a large population to be valid—investigating a small sample of a species, such as those individuals housed in one zoo, can lead to a greater understanding of that species and their needs. Mellen and MacPhee offered a framework for environment enrichment in 2001 involving Setting goals, Planning, Implementing, Documenting, Evaluating, and Readjusting (referred to as ‘SPIDER’). Since then, evaluation techniques have been highlighted in the species-specific context of cheetahs [64], lemurs [65] and lizards [66], and for training animals [67]. Alligood and Leighty [68] discuss different trends and the UK organisation National Centre for the Replacement, Refinement and Reduction of Animals in Research (NC3Rs) offers a valuable husbandry guide for researchers [69].
8. Finally, everything we learn about a small sample of a species housed in a zoo will inform research and conservation projects with its wild counterparts and have relevance to similar animals in other contexts. For example, in domestic environments, many rodents are frequently kept as companion animals. Mice, chinchillas, rats and hamsters are all nocturnal, very sensitive to light and noise, and usually active at night and around dawn and dusk. They need safe places to hide since they are prey animals, and they are highly sociable in the wild (except for Syrian hamsters). The RSPCA offers guidance on how best to look after rodents [70], but there is minimal legislation to ensure that pet owners treat their animals responsibly and provide appropriate welfare. Zoos offer an ideal opportunity to share information about nocturnal species with the wider public. Moreover, zoos’ commitment to conservation, research, visitor education and the wellbeing of the species they house has the potential to make a global impact in a wide range of contexts for both wild and managed animals.

## 6. Conclusions

The lives of nocturnal and crepuscular animals in nature and in managed care have long been a mystery to scientific observers, caregivers and nature lovers. Their activities have been obscured from our human senses in darkness. Today, new and upgraded technologies such as infrared and motion-activated sensors, recorders, and cameras, coordinated with RFID identification, tracking and imaging systems, and Animal-Computer Interaction programs are improving our understanding of how nocturnal species live in their wild and managed environments and what they need to thrive. This information can be transferred into action to support good animal welfare and public display possibilities in captive environments. Technology-assisted husbandry and enrichment can be used as an interdisciplinary approach to benefit the wellbeing of nocturnal species whilst simultaneously educating human stakeholders on species’ behaviour and ecology. Managed environments of nocturnal species may particularly benefit from artificial lighting and cameras as resources that permit and monitor the natural circadian cycle of nocturnal species, both when human carers are around and when they are not. Alongside technology, the continuing acquisition of new animal knowledge from wild research, evidence-based animal management, more detailed animal welfare assessments, and animal-centred habitat design frameworks, all suggest exciting opportunities to meet the needs of nocturnal species.

We explored some of these issues and techniques using the Moon Jam workshop process that brought together diverse participants to discuss and reflect on real challenges for nocturnal species housed in zoos. Workshop outcomes showed that a greater understanding of both species and individual animal senses, needs, preferences and motivations quickly led to testable concepts for improving nocturnal environments and offering enriching opportunities. Some were simple, such as potto arm-length sleeve feeders. Others could use existing commercial technology such as RFID smart pet gates and feeders. Still, others would require advanced technical design and testing. However, with the integration of Wi-Fi timers and applications synchronising weather data to outlet controls, these intricate systems may become as straightforward as downloading the appropriate app to manage all functionalities conveniently in the palm of one’s hand. This user-friendly approach is certainly foreseeable soon. 

The use of newer technology to enhance visitor experiences was not discussed as much in the workshop, but wearable infrared and starlight visors presently in use by the military and hunters could be adapted for use in zoos, sanctuaries and aquariums. Live infrared projections could reveal hidden animal activities, and now some smartphone cameras are adapted for extreme low-light conditions. Improved nocturnal habitats and management programs are likely to increase animals’ natural activity, with the potential for improving both the health and wellbeing of the animals and the engagement of visitors. 

In addition, rapid advancements in artificial intelligence techniques enable automated surveillance of animals. This decreases the need for human involvement, which can be stressful for other species, and also has the potential to offer positive welfare benefits. Machine Learning is being widely used to enable AI systems to recognise patterns in collections of data, therefore automating processes that previously required a human to spend a significant amount of time undertaking observations and analysing video recordings. Examples include (i) enhanced diagnoses of health conditions, (ii) the ability to monitor and interpret social behaviours and group dynamics and (iii) longitudinal passive data collection and analysis, to investigate seasonal variation. Moreover, facial or body recognition of individuals can enable the automation of bespoke feeding arrangements and access to areas of an enclosure, removing the requirement to tag animals. 

While managed nocturnal animals were our focus, essential knowledge we used was gained in field studies. Continued threats to wild nocturnal species globally, namely habitat loss and climate change, signify the growing importance of improving the lives of managed animals such as through developing animal-centred technology to safeguard these species. Moreover, by overcoming the current limitations of managed environments, there may be more scope to support the foundations of zoo, sanctuary and aquarium-based breed-and-release programs for rewilding endangered nocturnal species to suitable protected areas in the future.

## Data Availability

The original contributions presented in the study are included in the article, further inquiries can be directed to the corresponding author/s.
